# Hypertonic saline protects brain endothelial cells against hypoxia correlated to the levels of estimated glomerular filtration rate and interleukin-1β: Erratum

**DOI:** 10.1097/MD.0000000000006653

**Published:** 2017-04-07

In the article “Hypertonic saline protects brain endothelial cells against hypoxia correlated to the levels of estimated glomerular filtration rate and interleukin-1β”,^[1]^ which appeared in Volume 96, Issue 1 of *Medicine*, the title was given incorrectly. The correct title is as follows: Hypertonic saline protects brain endothelial cells against hypoxia correlated to the levels of epidermal growth factor receptor and interleukin-1β.
